# Malaria entomological surveillance in Kinshasa Province: A longitudinal study

**DOI:** 10.1371/journal.pgph.0006984

**Published:** 2026-08-05

**Authors:** Fabien Vulu, Melchior M. Kashamuka, Kristin Banek, Ruthly François-Zafka, Pitshou Mampuya, Joseph Atibu, Michael Emch, Victor Mwapasa, Jonathan J. Juliano, Jonathan B. Parr, Rhoel R. Dinglasan, Seth R. Irish, Antoinette K. Tshefu, Thierry L. Bobanga

**Affiliations:** 1 Department of Tropical Medicine, University of Kinshasa, Kinshasa, Democratic Republic of the Congo; 2 Institute for Global Health and Infectious Diseases, University of North Carolina at Chapel Hill, Chapel Hill, North Carolina, United States of America; 3 Ecole de Santé Publique, University of Kinshasa, Kinshasa, Democratic Republic of the Congo; 4 Department of Public Health, Kamuzu University of Health Sciences (KUHeS), Blantyre, Malawi; 5 Department of Epidemiology, Celia Scott Weatherhead School of Public Health and Tropical Medicine, Tulane University, New Orleans, Louisiana, United States of America; 6 Department of Epidemiology, Gillings School of Global Public Health, University of North Carolina at Chapel Hill, Chapel Hill, North Carolina, United States of America; 7 Department of Infectious Diseases and Immunology, College of Veterinary Medicine, University of Florida, Gainesville, Florida, United States of America; 8 Swiss Tropical and Public Health Institute (Swiss TPH), Allschwil, Switzerland; University of Nevada Las Vegas, UNITED STATES OF AMERICA

## Abstract

Malaria remains a major public health challenge in the Democratic Republic of Congo. To address the lack of contemporary entomological data, this four-year study characterized key entomological parameters in Kinshasa Province. We conducted a prospective observational cohort study (2018–2022) in three health areas. Adult mosquitoes were sampled using CDC light traps and pyrethrum spray catches. Sporozoite rates were estimated using ELISA, and trap collections were used as proxy measures of host-seeking mosquito activity to estimate light trap rates and annualized entomological inoculation rate (EIR) estimates. Insecticide susceptibility of adult females was assessed using WHO tube tests. A generalized estimating equations model evaluated the effects of key variables on mosquito abundance. A total of 1,449 *Anopheles* were collected, with 50% from rural, 49% from peri-urban, and 1% from urban areas. The species captured were *An. gambiae* s.l. (85%), *An. paludis* (11%), and *An. funestus* (4%). *An. gambiae* s.l. was significantly more abundant indoors and after 10 PM. The overall EIR estimate was 4.7 infective mosquitoes per trap per year, with *An. gambiae* s.l. contributing the highest transmission intensity. A localized increase in the transmission intensity associated with *An. paludis* was observed in one site during the rainy season, although mosquito numbers were small. Resistance to DDT and pyrethroids was widespread, with an estimated 0.94 frequency of the *kdr* L1014F mutation. Bendiocarb and pirimiphos-methyl remained effective. This study found that *An. gambiae* s.l. remains the predominant malaria vector in Kinshasa. The findings also suggest a possible contribution of *An. paludis* to malaria transmission in some rural settings, although additional longitudinal studies with larger sample sizes are needed. Because sporozoite detection was based on ELISA, additional molecular confirmation studies would be valuable. Continued entomological surveillance is essential to track biting behavior and resistance patterns and ensure that selected interventions remain effective.

## Background

Malaria remains a major public health challenge in the Democratic Republic of Congo (DRC), which bears the second highest malaria burden, with an estimated 12.5% of global malaria cases and 11.1% of deaths in 2025 [1]. Kinshasa Province presents a unique setting where varying levels of urbanization, landscape changes, and rapid population growth create diverse ecological conditions for malaria transmission. The province contains a mix of rural, peri-urban, and urban environments, each of which may influence vector behavior and malaria transmission dynamics [2].

Fundamental entomological data on malaria transmission remain insufficient and are often outdated or fragmented [1,3–8]. This lack of comprehensive and continuous entomological surveillance hinders efforts to understand local transmission patterns, vector species distribution, insecticide resistance, and the effectiveness of vector control measures. These knowledge gaps make it difficult to select and target effective vector control interventions in Kinshasa Province, where the malaria burden varies considerably across ecological settings [9,10].

Long-lasting insecticidal nets (LLINs) remain the primary malaria vector control tool in Kinshasa and across the DRC [11]. However, LLINs primarily target endophilic *Anopheles* mosquitoes, particularly *Anopheles gambiae* s.l., the principal malaria vector in the DRC, which predominantly bites at night, with peak activity occurring between 10 PM and 5 AM [1,12]. However, LLINs may not be fully protective against mosquitoes that bite earlier in the evening, bite outdoors or exhibit behavioral adaptations that reduce contact with LLINs [13,14]. Moreover, increasing insecticide resistance and changes in vector behavior threaten malaria control efforts in the DRC [6,8].

This study was conducted as part of a broader project integrating epidemiological, parasitological, and entomological approaches to improve understanding of malaria transmission across diverse ecological landscapes over time at seven sites spanning rural, peri-urban, and urban settings in Kinshasa Province [9]. Herein, we characterize the malaria entomological parameters including mosquito abundance, species composition, biting behavior, insecticide susceptibility, and resistance across diverse sites over four years.

## Methods

### Study sites

**Fig 1:**
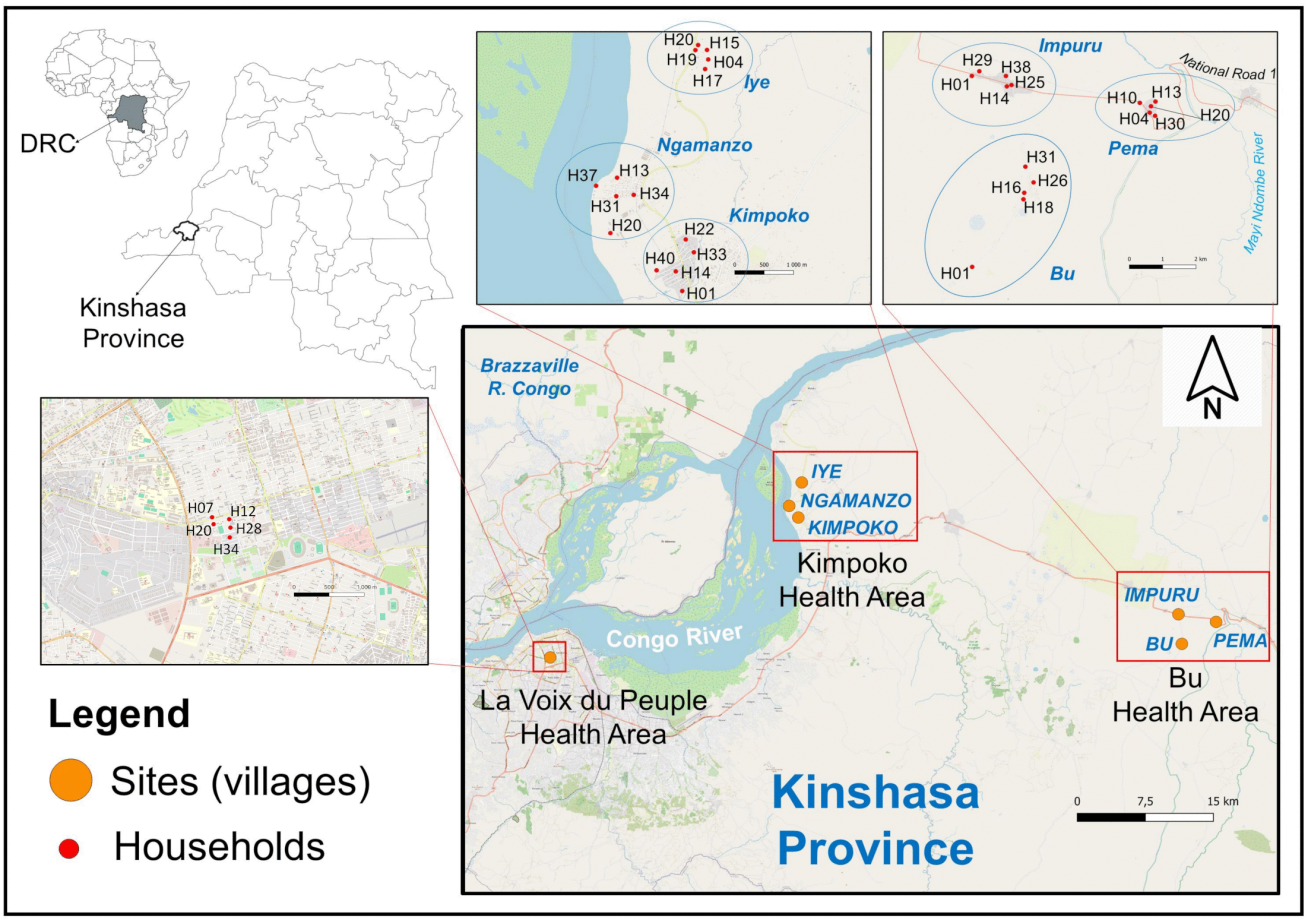
Location of study sites and households in Kinshasa Province, Democratic Republic of the Congo. Sites represented distinct transmission settings: Bu (rural, high transmission), Kimpoko (peri-urban, moderate transmission), and La Voix du Peuple (urban, low transmission). Household locations were jittered to protect participant confidentiality. The site map was created using data from OpenStreetMap under the Open Database License(https://www.openstreetmap.org/copyright).

From 2018 to 2021, we conducted a prospective observational cohort study across seven sites within three health areas (HAs) of Kinshasa Province: Bu (three sites: Bu, Impuru and Pema sites), Kimpoko (three sites: Kimpoko, Ngamanzo and Iye sites), and La Voix du Peuple (one site of the same name) (Fig 1). HAs are the smallest operational geographic units of the national health system in the DRC and are typically centered around a primary health care facility serving a defined population within a health zone, usually ranging from approximately 5,000–15,000 inhabitants [15]. These HAs were selected to represent the distinct ecological settings and malaria transmission intensities previously identified in Kinshasa Province [9,16]. Bu: Located on the Bateke Plateau within the Maluku 1 health zone, this rural, high-transmission area is marked by ancient sand dunes, expansive grasslands, patches of wooded savanna, narrow strips of dense gallery forest, and two river valleys. Within this HA, the Bu site sits approximately 3 km inland from National Road 1, whereas Impuru and Pema lie directly along the road. Kimpoko: Also located within the Maluku 1 health zone, this peri-urban, moderate-transmission area lies inside the swampy Maluku corridor along the Congo River. Within this HA, the Ngamanzo site sits directly on the riverbank, characterized by abundant aquatic vegetation and a small river port, while the other sites are located farther inland. La Voix du Peuple: Located in the Lingwala health zone, this urban, low-transmission area is a densely populated setting characterized predominantly by residential and commercial infrastructure, paved roads, and open drains (Fig 1). Kinshasa Province experiences a tropical humid climate, characterized by a nine-month rainy season from September to May and a three-month dry season from June to August. Mosquito collections occurred in both the rainy and dry seasons. The entomological household sampling methodology has been described previously [9]. In brief, a total of 35 households (five per site) were randomly selected to ensure a spatially representative coverage, and 32 households remained enrolled at the final visit.

### Adult mosquito collection and morphological identification

We conducted nine rounds of adult *Anopheles* mosquito collection using U.S. Centers for Disease Control and Prevention Miniature Light Traps (CDC LTs) (John W. Hock Company, model 512) and pyrethrum-spray catches (PSC). Three rounds of mosquito collection took place in both 2018 and 2019, two rounds in 2020, and one round in 2021 (S1 Table) [9]. Due to COVID-19 restrictions during 2020 in the urban site (Voix du Peuple HA), the seventh and eighth mosquito collection rounds were not conducted. However, all sites participated again during the final (ninth round) visit in 2021.

Two CDC LTs were set per house for three consecutive nights; one indoors near occupied sleeping spaces protected by bed nets and one outdoors near the household entrance; to sample host-seeking mosquitoes. Traps were set at 6 PM, then emptied and reset at 10 PM. The traps set at 10 PM were emptied the following morning at 6 AM. The timing of the trap setting was informed by preliminary discussions with heads of households indicating that children and adults went to bed between 6 – 8 PM and 8 – 10 PM, respectively. Analyses comparing indoor versus outdoor collections, as well as those before 10 PM versus after 10 PM, were based exclusively on CDC LT data.

After three nights of trapping, PSCs were performed in the houses using a pyrethroid-based commercial insecticide to capture indoor-resting mosquitoes. PSCs were also used to improve the characterization of species composition across study sites by complementing CDC LT collections. PSCs were conducted starting at 6 AM in the morning following the third night of CDC LT collections, before residents opened household doors and windows and prior to residents beginning their daily activities. Only one sleeping room per household was selected for PSC collection, corresponding to the room in which the indoor CDC LT had been installed. Household inhabitants (humans and animals), as well as food items, water, and cooking utensils, were temporarily moved at least 30 meters away from the house before spraying. White sheets were spread across the floor to fully cover the room’s surface, and openings in the room were sealed as much as possible. A pyrethroid-based aerosol insecticide was then sprayed until a fine mist was obtained. After 30 minutes, the white sheets containing knocked-down mosquitoes were carefully removed and taken outdoors for mosquito collection under natural daylight conditions.

Upon collection in the field, *Anopheles* mosquitoes were separated from other mosquito genera (*Culex*, *Aedes*, and *Mansonia*) based on morphological characteristics and placed individually in 1.5 mL microtubes containing silica gel. Tubes were labeled according to collection method, household, site, and collection period. Samples were then transferred to the insectary of the Department of Tropical Medicine at the University of Kinshasa for morphological species identification using Gillies and De Meillon’s keys and stored at –4 °C for further analyses [17,18].

### Larval collection and insecticide bioassays

Collections of *Anopheles* aquatic stages (larvae and pupae**)** were conducted on two separate occasions in May 2019 and February 2022 in the Bu and Kimpoko HAs. These periods corresponded to the rainy season when *Anopheles* larval abundance was expected to be high. Each collection period lasted approximately 3–7 days, depending on the time required to obtain sufficient numbers of adult mosquitoes from the collected larvae for downstream analyses. Breeding sites consisted mainly of small, sun-exposed rainwater pools typically associated with *An. gambiae* s.l. ecology. The same larval collection sites were revisited during subsequent collection. Larval habitats were sampled opportunistically during each collection period; however, the total number of habitats and larvae collected was not systematically recorded because the objective was not to estimate larval density. We did not collect *Anopheles* aquatic stages in the La Voix du Peuple HA because of the paucity of *Anopheles* mosquitoes in this HA. Upon collection, larvae and pupae were taken to the field insectaries and reared to the adult stage. These insectaries were set up in the primary health centers of Bu and Kimpoko using basic equipment which included larval rearing trays and adult mosquito cages. Temperature and humidity were monitored four times a day and during assays using digital devices and remained within the recommended ranges for all assays [19].

To assess insecticide resistance status of *Anopheles* mosquitoes, we conducted WHO susceptibility tube tests on three- to five-day-old, non-blood-fed F0 adult female *Anopheles* mosquitoes [19]. For each insecticide test, four replicates of 20–25 females fed *ad libitum* on a 10% sugar solution were exposed for one hour to paper impregnated with DDT 4% (Dichlorodiphenyltrichloroethane, organochlorine), deltamethrin 0.05% (pyrethroid), permethrin 0.75% (pyrethroid), alphacypermethrin 0.05% (pyrethroid), pirimiphos-methyl 0.25% (organophosphate) or bendiocarb 0.1% (carbamate). For the control tests, two replicates of 20–25 adult female mosquitoes were exposed to untreated papers. During the 24-hour post-exposure observation period, mosquitoes were fed ad libitum on a 10% sugar solution. Both the insecticide-impregnated and control papers were purchased from Universiti Sains, Malaysia.

Mosquito mortality was recorded 24 hours after exposure. Resistance status was interpreted according to WHO criteria [18]. Mortality rates between 98% and 100% were considered indicative of susceptibility, mortality rates between 90% and 97% indicated possible resistance that required confirmation, and mortality rates below 90% were considered indicative of confirmed resistance. For population showing possible resistance, resistance was considered confirmed only if repeat testing on a new mosquito sample again produced mortality below 98% [18].

### Molecular identification of sibling species of the *Anopheles gambiae* complex

DNA was extracted from the wings and legs of adult-caught *An. gambiae* s.l. following a CTAB (Cetyltrimethylammonium Bromide) technique adapted by the Molecular Biology Unit of the National Institute of Biomedical Research (INRB) (S1 Text). PCR assays were conducted using the F6 and R5 primers developed by Salako et al. [20], which were adapted from those used by Santolamazza et al. [21] to identify three species of the *An. gambiae* complex (*An. gambiae* s.s.*, An. coluzzii, and An. arabiensis*), including hybrid individuals (S2 Table). Only a subset of morphologically identified *Anopheles* mosquitoes was analyzed by PCR because of resource and reagent limitations; the remaining specimens were not processed further for molecular identification. In addition, specimens collected from La Voix du Peuple were excluded from molecular analysis due to the very low number (n = 12) of *Anopheles* mosquitoes sampled at this urban site.

### Sporozoite rate

To determine the sporozoite rate (SR), enzyme-linked immunosorbent assays (ELISA) were performed on field-collected adult female *Anopheles* mosquitoes. The head–thorax portion of each mosquito was separated and individually homogenized in phosphate-buffered saline containing 0.05% Tween 20 (PBS-T). The homogenates were processed following a modified Wirtz protocol, using the CDC ELISA reagent kit for circumsporozoite protein detection (BEI Resources, NIAID, NIH, USA) [22,23]. Plates were blocked with 1% bovine serum albumin (BSA) in PBS-T. The ABTS substrate (2,2’-Azino-bis (3-ethylbenzothiazoline-6-sulfonic acid)) was prepared using Peroxidase Substrate Solution B (KPL, 450 mL, 910 Clopper Road, Gaithersburg, MD, USA) and phenol red sodium salt (Sigma-Aldrich) for color development. All incubations were carried out at 37 °C. Optical density (OD) was measured at 405 nm after 30 min using an 800 TS microplate reader (BioTek). A sample was considered positive if its OD value was at least twice the mean OD of the negative controls [24].

### Knockdown resistance mutation genotyping

To detect the knockdown resistance (*kdr*) L1014F gene mutation in the sodium channel associated with resistance to DDT and pyrethroids in *Anopheles* mosquitoes, DNA was extracted from the wings and legs of adult-caught *Anopheles* mosquitoes as described above. Genotyping was performed on adult-caught *Anopheles* specimens using the *KDR*-Li protocol proposed by Huynh et al. [25]. We restricted *kdr* genotyping to *An. gambiae* s.l. because this species complex was the predominant vector identified in our study sites and the L1014F mutation is the most extensively characterized resistance marker in malaria vectors from the DRC [6,26]. While this mutation is well-documented in *An. gambiae* s.l., recent studies have nevertheless shown that resistance-associated mutations affecting the voltage-gated sodium channel are also present in other mosquito species and genera, including *An. funestus* [27–29].

### Statistical analysis

Data were analyzed using R version 4.4.1. Descriptive statistical analyses were conducted to evaluate the abundance of *Anopheles* species across host-seeking locations (indoor vs. outdoor), time periods, collection sites, HAs, seasons, and years. Chi-square and Fisher’s exact tests were employed to assess associations between categorical factors and mosquito species. For comparisons involving continuous variables, the Mann-Whitney U test was applied. Statistical significance threshold was set at 0.05.

We evaluated the associations between multiple factors (year, season, site, capture location, and time) and the abundance of *Anopheles* spp. using Generalized Estimating Equations (GEE) regression models. The goal was to account for the correlation between observations within clusters defined by study HA. Analyses were conducted using the geepack (version 1.3.12), MASS (version 7.3.60.2), and broom (version 1.0.7) R packages.

Because mosquito count data showed evidence of overdispersion, preliminary negative binomial models were first explored. Overdispersion was assessed by comparing the residual deviance to the residual degrees of freedom and by examining dispersion parameters obtained from these preliminary models. However, final analyses were conducted using GEE models with a Poisson distribution, corrected for overdispersion using the dispersion parameter (θ) estimated from the preliminary negative binomial models. This approach was selected because GEE models allowed appropriate handling of correlated observations within clusters while providing robust, population-averaged estimates.

An exchangeable correlation structure was selected because observations collected within the same cluster were assumed to be similarly correlated. Model adequacy and correlation structure selection were assessed using the Quasi-likelihood under the Independence model Criterion (QIC), which is appropriate for GEE models because traditional likelihood-based criteria such as, the Akaike Information Criterion (AIC) are not directly applicable [30]. Lower QIC values were used to guide the selection of the final model and correlation structure. Wald tests were used to assess the statistical significance of model parameters. Missing values (for capture location and collection period) for PSC-collected mosquitoes were handled using weighted frequency imputation, whereby missing values were replaced proportionally based on the observed distribution of each category within the variable.

To estimate relative host-seeking activity, CDC LTs were used as indirect proxies for human exposure to mosquito bites. While this method can either underestimate or overestimate the actual risk of malaria transmission to humans [31–33], several studies have demonstrated the reliability and validity of using CDC LTs to estimate the light trap rate (LTR) [34,35]. The LTR was calculated by dividing the number of *Anopheles* mosquitoes captured in a specific area by the number of traps and trapping nights, yielding the number of mosquitoes collected per light trap per night (mosquito/LT/night), used as an indirect proxy for human exposure to mosquito bites.

The SR was calculated as the number of mosquitoes testing positive for *Plasmodium* sporozoites by ELISA, over the total number of mosquitoes tested. Only mosquitoes collected using CDC LTs were included in calculation of SR and the trap-derived entomological inoculation rate (EIR) to maintain methodological consistency between host-seeking mosquito collections and transmission estimates. The trap-derived EIR was determined by multiplying the LTR by the SR and then by 365, providing an approximate annualized estimate of potential malaria transmission intensity:


$$\begin{array}{c} \text{EIR }=\text{ LTR }\times \text{ SR }\times \;\text{3}\text{6}\text{5} \end{array}$$

where EIR represents the estimated annual number of infected mosquitoes per trap per year based on CDC LT collections.

The frequencies of the *kdr* L1014F mutations were calculated using the following formula:


$$\begin{array}{c} \text{F}(kdr)\;=\;(\text{2}\text{RR}+\text{RS})/\text{2}\text{n} \end{array}$$

where RR represents the number of mosquitoes homozygous for the resistant allele, RS represents the number of heterozygous individuals, and n is the total number of tested individuals.

### Ethics approval and consent to participate

The study protocol was approved by the Ethics Committee of the Kinshasa School of Public Health, University of Kinshasa (Approval No. ESP/CE/021/2017), and by the Institutional Review Board of the University of North Carolina at Chapel Hill (Approval No. 17–1588). Authorization to conduct field activities and access study sites was obtained from the Provincial and Health Zone authorities. Community leaders (traditional chiefs and elders) were consulted in each study site before the study began. Written informed consent was obtained in Lingala or French from participants aged at least 17 years and older. For participants younger than 17 years, parental or guardian consent was obtained. Additionally, assent was obtained from participants aged 7–17 years prior to data collection.

## Results

### *Anopheles* abundance

Between March 2018 and March 2021, a total of 1,449 *Anopheles* mosquitoes were collected from 35 participating households. The majority of mosquitoes (n = 1,288; 88.8%), were captured using CDC LTs. Among these, 937 (72.7%) were collected indoors and 856 (66.4%) were caught after 10 PM, irrespective of the collection location. Overall, 1,232 (85.0%) of all mosquitoes were collected during the rainy season. Nearly all specimens in this study were obtained from the rural HA of Bu (n = 721, 49.7%) and the peri-urban HA of Kimpoko (n = 716, 49.4%). By site, the highest abundance of *Anopheles* mosquitoes was recorded in Bu (n = 548, 37.8%) and Kimpoko (n = 531, 36.6%). In contrast, La Voix du Peuple (n = 12, 0.8%) and Pema (n = 37, 2.5%) had the lowest abundance.

### *Anopheles* species

**Fig 2:**
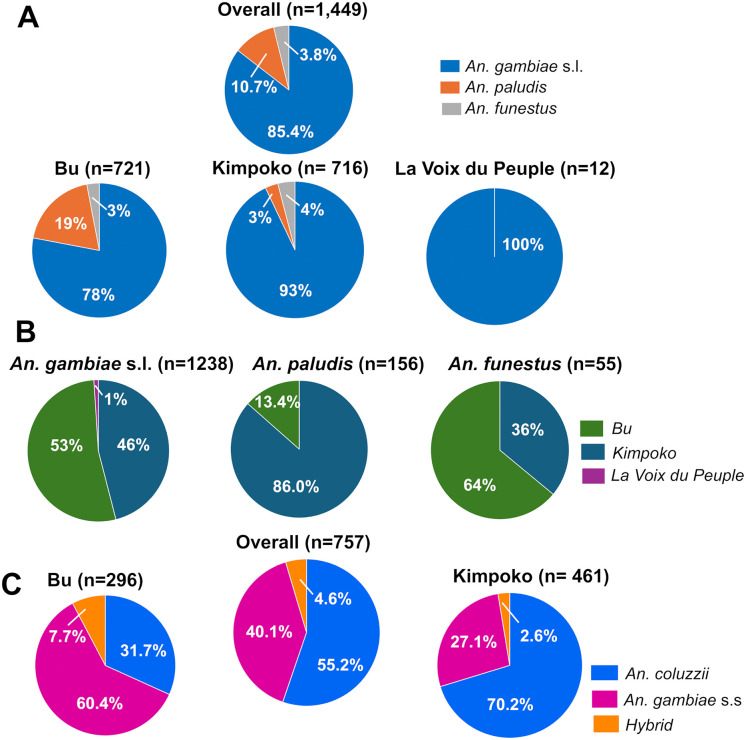
Distribution of *Anopheles* species. (A. Abundance of *Anopheles* by health area. **B.** Distribution of *Anopheles* species across each health area. **C.** *An. gambiae* s.l. sibling species abundance in each area). Note: Sibling species testing was not performed for La Voix du Peuple due to the limited number (n = 12) of *An. gambiae* s.l. specimens collected there.

Three *Anopheles* mosquito species were detected: *An. gambiae* s.l. (n = 1,238; 85.4%), *An. paludis* (n = 156; 10.7%), and *An. funestus* (n = 55; 3.8%) (Fig 2A). The species distribution varied significantly across the HA (Fisher’s exact test, p < 0.001). Specifically, 53.3% (n = 660) of *An. gambiae* s.l. were collected in the Kimpoko HA and 44.9% (n = 556) in the Bu HA. In contrast, 86.5% (n = 135) of *An. paludis* specimens were found in the Bu HA, while 63.6% (n = 35) of *An. funestus* were recorded in Kimpoko HA. In the La Voix du Peuple HA, only *An. gambiae* s.l. was recorded (Fig 2B, S3 Table).

**Fig 3:**
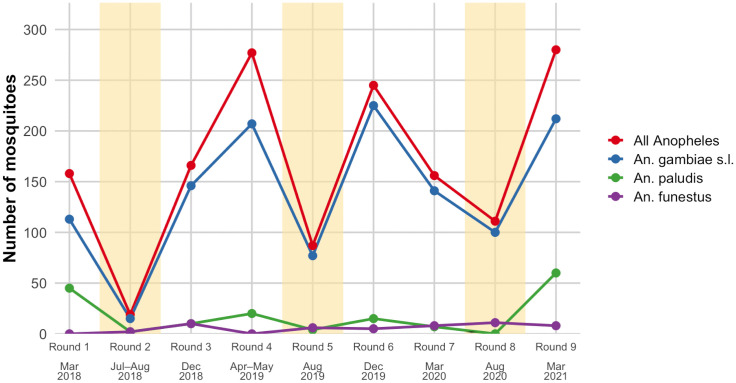
Abundance of *Anopheles* mosquito species over the study period.

The number of *Anopheles* species varied across the different collection rounds, showing notable fluctuations between seasons (Fig 3). Median counts were 141 for *An. gambiae* s.l., 10 for *An. paludis*, and 8 for *An. funestus*. Abundances were significantly higher for *An. gambiae* s.l. and *An. paludis* during the rainy season (both p = 0.03), whereas no seasonal difference was observed for *An. funestus* (p = 1.0).

Of the 1,238 *An. gambiae* s.l. specimens captured in this study, 757 were molecularly identified to the sibling species level. Among these, 418 (55.2%) were identified as *An. coluzzii*, 304 (40.1%) as *An. gambiae* s.s., and 35 (4.6%) as hybrids of *An. coluzzii* and *An. gambiae* s.s. No *An. arabiensis* specimens were detected. The composition of *An. gambiae* s.l. sibling species varied significantly across HAs (χ² test, p < 0.001) (Fig 2C).

### *Anopheles* behavior

The majority of mosquitoes were captured indoors (n = 937), with 667 collected after 10 PM and 270 collected before 10 PM. In contrast, fewer numbers were collected outdoors (n = 351), with 189 after 10 PM and 162 before 10 PM. Significant differences in mosquito abundance were observed by location (indoor vs outdoor) (IRR: 2.1 [95% CI: 1.4–3.1], p = 0.003) and period (before vs after 10 PM) (IRR: 1.7 [95% CI: 1.2–2.5], p = 0.004) (Tables 1 and 2). When analyzed by species, *An. gambiae* s.l. was the predominant species, showing a significantly higher abundance both indoors (p = 0.01) and after 10 PM (p = 0.02). No significant differences in host-seeking behavior were found for *An. paludis* or *An. funestus* (Table 1).

**Table 1:** *Anopheles* species host-seeking behavior.

Species	Capture location		P	Capture period		p
	Outdoor	Indoor		Before 10 PM	After 10 PM	
***An. gambiae* s.l.**	Me: 30 (IQR: 14–47)	Me: 80 (IQR: 62–130)	0.01*	Me: 37 (IQR: 23–47)	Me: 77 (IQR: 64–117)	0.02*
	n = 264 (24.3%)	n = 821 (75.6%)		n = 312 (28.7%)	n = 773 (71.2%)	
***An. paludis***	Me: 4.5 (IQR: 1–12.5)	Me: 5 (IQR: 2–9)	0.68	Me: 4 (IQR: 2–11)	Me: 5 (IQR: 1–6)	0.72
	n = 68 (44.4%)	n = 85 (55.5%)		n = 98 (64.0%)	n = 55 (35.9%)	
***An. funestus***	Me: 0 (IQR: 0–3)	Me: 3 (IQR: 1–6)	0.27	Me: 2 (IQR: 1–4)	Me: 4 (IQR: 1–5)	0.53
	n = 19 (38%)	n = 31 (62%)		n = 22 (44%)	n = 28 (56%)	

Me: Median number of mosquitoes collected per round; IQR: Interquartile range, n = Total number of specimens from all rounds *Statistically significant (*Mann–Whitney U* test).

**Table 2:** Factors associated with *Anopheles* abundance.

Variable	Estimate (β)	SE	IRR	IRR 95% CI	p
Year: 2018	Reference				
Year: 2019	0.5	0.2	1.7	1.0–2.8	0.04*
Year: 2020	0.1	0.3	1.1	0.6–2.0	0.61
Year: 2021	0.8	0.2	2.3	1.4–3.7	<0.001*
Season: Dry	Reference				
Season: Rainy	0.8	0.3	2.3	1.3–4.4	0.01*
Site: La Voix du Peuple	Reference				
Site: Bu	2.2	0.3	9.5	5.2–17.2	<0.001*
Site: Impuru	0.8	0.4	2.3	1.1–4.9	0.03*
Site: Iye	1.0	0.3	2.9	1.5–5.5	<0.001*
Site: Kimpoko	2.4	0.3	11.3	6.4–19.8	<0.001*
Site: Ngamanzo	0.9	0.2	2.4	1.5–4.1	0.001*
Site: Pema	0.7	0.4	2.1	0.9 – 5.0	0.08
Time: Before 10 PM	Reference				
Time: After 10 PM	0.5	0.2	1.7	1.2–2.5	0.004*
Location: Outdoor	Reference				
Location: Indoor	0.7	0.2	2.1	1.4–3.1	0.003*

SE: Standard Error, IRR: Incidence Rate Ratio, CI: Confidence Interval, *Statistically significant (GEE).

### Factors associated with *Anopheles* abundance

Overall, *Anopheles* abundance was higher in 2019 and 2021 compared to 2018, during the rainy season, indoors, and after 10 PM (Table 2). The Bu and Kimpoko sites had significantly higher mosquito abundance than La Voix du Peuple. *An. gambiae* s.l. abundance increased in 2019 and marginally in 2021 compared with the first collection year (2018). It was significantly higher in the rainy season, indoors, after 10 PM, and at all sites except Pema (Table 3). *An. paludis* was significantly less abundant in 2020, but more abundant during the rainy season and at Bu in particular. No association was found with location or collection period (Table 4).

**Table 3:** Factors associated with *Anopheles gambiae* s.l. abundance.

Variable	Estimate (β)	SE	IRR	IRR 95% CI	p
Year: 2018	Reference				
Year: 2019	0.7	0.2	1.9	1.1–3.4	0.01*
Year: 2020	0.3	0.3	1.4	0.7–2.7	0.32
Year: 2021	0.7	0.3	1.9	1–3.9	0.05
Season: Dry	Reference				
Season: Rainy	0.8	0.3	2.4	1.2–4.7	0.01*
Site: La Voix du Peuple	Reference				
Site: Bu	2.3	0.3	10.5	5.6–19.8	<0.001*
Site: Impuru	1.1	0.4	2.9	1.3–6.9	0.01*
Site: Iye	1.2	0.3	3.2	1.7–6.0	0.003*
Site: Kimpoko	2.7	0.3	14.8	8.0–27.4	<0.001*
Site: Ngamanzo	0.9	0.3	2.6	1.5–4.5	<0.001*
Site: Pema	0.7	0.5	2.1	0.8–5.3	0.12
Time: Before 10 PM	Reference				
Time: After 10 PM	0.7	0.2	2.145	1.5–3.0	<0.001*
Location: Outdoor	Reference				
Location: Indoor	0.9	0.2	2.616	1.7–4.0	<0.001*

SE: Standard Error, IRR: Incidence Rate Ratio, CI: Confidence Interval, *Statistically significant (GEE).

**Table 4:** Factors associated with *Anopheles paludis* mosquito abundance.

Variable	Estimate (β)	SE	IRR	IRR 95% CI	p
Year: 2018	Reference				
Year: 2019	-0.4	0.6	0.6	0.1–2.1	0.47
Year: 2020	-1.5	0.6	0.2	0.1–0.7	0.02*
Year: 2021	0.8	0.6	2.2	0.7–7.2	0.19
Season: Dry	Reference				
Season: Rainy	1.4	0.5	4.0	1.3–12.0	0.01*
Site: Iye	Reference				
Site: Bu	2.6	0.56	12.8	4.0–40.9	<0.001*
Site: Impuru	1.0	0.7	2.7	0.7–10.3	0.13
Site: Kimpoko	0.6	0.7	1.5	0.4–6.2	0.51
Site: La Voix du Peuple	-42.2	0.7	4 x 10^–19^	1 x 10^–19^–1 x 10^–18^	<0.001*
Site: Ngamanzo	-41.7	0.6	8 x 10^–19^	2 x 10^–19^–2 x 10^–18^	<0.001*
Site: Pema	-42.3	0.7	4 x 10^–19^	1 x 10^–19^–1 x 10^–18^	<0.001*
Time: Before 10 PM	Reference				
Time: After 10 PM	-0.7	0.4	0.5	0.2–1.0	0.06
Location: Outdoor	Reference				
Location: Indoor	0.2	0.4	1.2	0.5–2.7	0.61

SE: Standard Error, IRR: Incidence Rate Ratio, CI: Confidence Interval, *Statistically significant (GEE).

### Light trap rate, sporozoite rate, and trap-derived entomological inoculation rate

The overall LTR was 0.76 mosquito per light trap per night, with the highest rates observed in both Bu (0.83) and Kimpoko (0.81) HAs. *An. gambiae* s.l. had the highest species-specific LTR at 0.64. The overall SR was 1.71% (17/993 mosquitoes tested), with rates of 1.53% (6/393) in Bu and 1.86% (11/591) in Kimpoko HAs, while Pema sites recorded a site-specific SR of 11.11% (1/9) (Table 5). The annualized trap-derived EIR estimate was 4.74 infective mosquitoes per trap per year. This rate was higher in Bu (4.63) and Kimpoko (5.41), and zero in La Voix du Peuple (Table 5). Overall, EIR estimate was higher during the rainy season (5.91 vs 2.75), indoors (7.54 vs 1.87), and after 10 PM (6.82 vs 2.69). Although *An. gambiae* s.l. had the highest estimated species-specific EIR (4.01), *An. paludis* EIR peaked in Impuru during the rainy season (8.92).

**Table 5:** Entomological risk of malaria transmission.

Health Areas	Sites	LTR	SR	EIR estimate		
				Rainy season	Dry season	All
Bu	Bu	1.77	1.00	10.01	0.00	6.46
	Impuru	0.53	2.41	5.66	0.00	4.66
	Pema	0.13	11.11	8.29	0.00	5.27
	**Total**	**0.83**	**1.53**	**10.28**	**0.00**	**4.63**
Kimpoko	Kimpoko	1.81	1.74	11.23	11.72	11.49
	Ngamanzo	0.24	5.60	4.37	5.43	4.90
	Iye	0.37	0.00	0.00	0.00	0.00
	**Total**	**0.81**	**1.86**	**5.52**	**5.23**	**5.41**
La Voix du Peuple	La Voix du Peuple	0.05	0.00	0.00	0.00	0.00
**Total for all sites**		**0.76**	**1.71**	**5.91**	**2.75**	**4.74**
***Anopheles* species**						
*An. gambiae* s.l.		0.64	1.72	4.74	2.32	4.01
*An. paludis*		0.09	1.44	0.67	0.00	0.47
*An. funestus*		0.03	2.00	0.18	0.00	0.21
**Location**						
Outdoor		0.41	1.25	2.65	0.00	1.87
Indoor		1.11	1.86	14.02	2.99	7.54
**Collection Time**						
Before 10 PM		0.51	1.45	1.67	3.77	2.69
After 10 PM		1.01	1.85	9.27	2.29	6.82

LTR (Light Trap Rate): Mosquitoes per light trap per night; **SR** (Sporozoite Rate): %; **EIR** estimate (Entomological Inoculation rate): Infective mosquitoes per trap per year.

### Resistance status to insecticides

In Bu, resistance to DDT was observed in 2019, with a mosquito mortality rate of 25%. By 2022, this mortality rate had increased substantially to 89% (Table 6). In 2019, the mortality rates for deltamethrin and alphacypermethrin were 86% and 72%, respectively. Deltamethrin mortality remained similar over time, while alphacypermethrin showed a modest improvement, increasing from 72% in 2019 to 82% in 2022. Permethrin, tested only in 2022, showed evidence of resistance with a mortality rate of 55.4%. In contrast, both bendiocarb and pirimiphos-methyl remained fully effective in both years, achieving 100% mortality. In Kimpoko, strong resistance to DDT was also observed in 2019, but this improved markedly by 2022 (increasing from 9.7% to 92.7% mortality). Pyrethroid resistance varied: deltamethrin mortality improved from 76.3% in 2019 to 95.8% in 2022, while alphacypermethrin mortality remained stable. Bendiocarb and pirimiphos-methyl were consistently effective across both years, yielding 100% mortality.

**Table 6:** Insecticide susceptibility status of *Anopheles* mosquitoes in Bu and Kimpoko.

Insecticide type	Bu health area						Kimpoko health area					
	2019			2022			2019			2022		
	n	Mortality	Status	n	Mortality	Status	n	Mortality	Status	n	Mortality	Status
DDT 4%	80	25%	**R**	100	89%	**R**	80	9.7%	**R**	100	92.7%	**R**
Permethrin 0.75%	80	55.4%	**R**	100	NT	NT	80	54.2%	**R**	NT	NT	NT
Deltamethrin 0.05%	80	86%	**R**	100	86.3%	**R**	80	76.3%	**R**	100	95.8%	**R**
Alphacypermethrin 0.05%	80	72%	**R**	100	82.8%	**R**	80	87.8%	**R**	100	88.7%	**R**
Bendiocarb 0.1%	80	100%	**S**	100	100%	**S**	80	100%	**S**	100	100%	**S**
Pirimiphos-methyl	NT	NT	NT	100	100%	**S**	NT	NT	NT	100	100%	**S**

n = number of tested mosquitoes, S: Susceptible, R: Resistant, NT: Not tested.

### Markers of insecticide resistance (*kdr* mutation)

A total of 709 *An. gambiae* s.l. specimens were successfully analyzed for the presence of the *kdr* L1014F mutation, including 259 from Bu, 448 from Kimpoko, and 2 from La Voix du Peuple. Of these, 662 were homozygous resistant, 16 were heterozygous, and 31 were homozygous susceptible (wild-type). The overall frequency of the L1014F mutation was high (0.944), with slightly lower values in Bu (0.918) than in Kimpoko (0.959) (p = 0.04). Mutation frequencies were similar across sibling species: *An. coluzzii* (0.949), *An. gambiae* s.s. (0.940), and hybrids (0.913) (p = 0.81). This high frequency is reflected by the fact that the majority of specimens (n = 662; 93.3%) were homozygous for the resistant allele.

## Discussion

This study reveals three key findings with direct implications for malaria control in Kinshasa Province. First, *An. gambiae* s.l. remains the dominant malaria vector, exhibiting high indoor and late-night biting activity, which supports the continued use of LLINs. However, the data also reveal specific gaps in protection, as some *An. gambiae* s.l. subpopulations exhibited early-evening biting (before 10 PM) and outdoor biting behavior, which may reduce the effectiveness of LLINs. Second, despite regional variations in transmission risk, the comparable trap-derived EIR found in the rural (Bu), and peri-urban (Kimpoko) areas indicate that malaria transmission remains robust in both settings. These entomological findings converge with epidemiological and parasitological analyses from the same cohort study, which also highlighted sharp spatial disparities in malaria burden. Specifically, while urban areas experienced a very low burden, transmission was substantially higher in both peri-urban and rural settings, with the latter exhibiting the highest burden overall [9]. This suggests that interventions should not only be geographically tailored but also maintained at scale across both rural and peri-urban zones. Third, widespread resistance to pyrethroids and near-fixation of the L1014F *kdr* mutation threaten the efficacy of current LLINs, although the preserved susceptibility to bendiocarb and pirimiphos-methyl provides valuable alternatives for complementary control tools such as indoor residual spraying (IRS). These findings should nevertheless be interpreted cautiously because mosquito sample sizes were limited in certain sites, collection efforts varied across HAs, and EIR estimates relied on CDC LTs. Collectively, these findings underscore the importance of sustaining malaria control through LLINs. However, long-term effectiveness will require adaptive, localized strategies, resistance-informed insecticide selection, ongoing entomological surveillance, and consideration of next-generation LLINs, including dual-action nets, in areas where pyrethroid resistance is well established.

The predominance of *An. gambiae* s.l. across all study sites confirms its central role as the primary malaria vector in Kinshasa, consistent with prior findings in both the province and the wider DRC [1,4,36–40]. Its strong preference for indoor, late-night biting supports current LLIN-based strategies [1,8,11,41–48]. Yet, while LLINs remain a cornerstone of malaria prevention, growing evidence suggests that their long-term effectiveness may be reduced in some settings due to insecticide resistance, physical net deterioration, and changing mosquito behaviors [6,13,44,49–51]. Furthermore, despite a low proportion, the occurrence of bites outdoors and before 10 PM highlights early and outdoor biting behavior, potentially allowing for residual transmission despite LLIN coverage. This vector behavior has been reported in other African settings [44]. Crucially, *An. gambiae* s.l. is a complex of sibling species with distinct ecological and behavioral traits [41,52–56]. Because this study identified *An. coluzzii*, *An. gambiae* s.s., and hybrid forms, the observed biting patterns may reflect the combined behaviors of multiple sibling species co-existing across the study areas. Consequently, future research must investigate species-specific behavioral dynamics within the *An. gambiae* complex to optimize targeted interventions. Beyond the primary vector, this study highlights the potential epidemiological importance of secondary species. Specifically, *An. paludis* accounted for approximately 11% of the total *Anopheles* population and contributed about 10% of the overall EIR estimate. This contribution was marked by a localized peak in Impuru during the rainy season, highlighting its possible role in seasonal and localized transmission. Although often considered a secondary vector, *An. paludis* exhibits exophagic and early-evening biting tendencies compared with *An. gambiae* s.l. [41,54]. These behaviors can facilitate outdoor or pre-bedtime transmission, thereby reducing the effectiveness of LLINs. In such settings, conventional tools alone may be insufficient, underscoring the need for context-specific vector surveillance and supplementary control measures, such as outdoor residual spraying and insecticide-impregnated screens [45]. However, these *An. paludis* estimates should be interpreted cautiously because they were derived from a relatively small number of tested mosquitoes and may therefore be sensitive to small variations in mosquito abundance and sporozoite positivity. Also, these EIR estimates were derived from CDC LT collections and should be interpreted as proxy estimates of relative malaria transmission intensity rather than direct measurements of human exposure. While CDC LTs are widely used for malaria vector surveillance, they may not fully reflect true human biting rates because trap attractiveness can vary according to mosquito species, density, environmental conditions, and indoor versus outdoor settings. Consequently, LTs may attract or divert host-seeking mosquitoes toward the trap, potentially influencing the observed biting patterns and mosquito abundance [31–35].

As previously mentioned, the two sibling species identified within the *An. gambiae* complex were *An. coluzzii* (55%) and *An. gambiae* s.s. (40%). *An. arabiensis* was absent, consistent with its typical restriction to eastern DRC [11,46–48]. This species distribution aligns with previous reports in Kinshasa [6,37,39,46–48]*. An. coluzzii* predominated in Kimpoko, likely due to its opportunistic breeding habits and preference for stable, semi-permanent water bodies. These conditions are favored by the proximity to the Congo River and the presence of marshlands, fishponds, and slow-flowing streams. Specifically, the Ngamanzo site, located on the riverbank and characterized by abundant aquatic vegetation and a small river port, provides ideal habitats for *An. coluzzii*, which tolerates moderate organic pollution and adapts well to anthropogenic environments [52,53]. In contrast, *An. gambiae* s.s. dominated in Bu. The breeding sites in Bu are primarily temporary, rain-fed, and less disturbed, aligning with the preference for *An. gambiae* s.s. for small, clean, sunlit water bodies with minimal vegetation and low organic content. These observations further illustrate the ecological divergence between these two sibling species [52,54–56]. Low hybridization rates were detected between the two sibling species, consistent with earlier findings [50]. Yet, the presence of these rare hybrid forms is noteworthy. Hybridization may facilitate gene flow between the species, potentially spreading adaptive traits such as insecticide resistance and behavioral mechanisms across ecological settings [52,54–56]. While this study did not investigate the functional consequences of hybridization, these findings highlight the importance of continued molecular surveillance to understand its evolutionary and epidemiological implications in malaria vector populations.

The high malaria transmission risk observed in Kimpoko and Bu is likely driven by favorable ecological conditions that support elevated mosquito densities [15,57,58]. In Kimpoko, environmental conditions such as the proximity to the Congo river, appear to sustain relatively stable transmission across both seasons. In contrast, Bu shows marked seasonal variation, with increased transmission risk during the rainy season, presumably due to the temporary availability of breeding habitats created by rainfall. The very low estimated transmission intensity observed in the urban La Voix du Peuple HA likely reflects the effects of urbanization, such as reduced breeding habitats. These patterns are consistent with a broader body of evidence from Kinshasa and other urban African settings showing that malaria prevalence and transmission intensity tend to decline with increasing urbanization, while peri-urban and rural areas often sustain higher transmission because of greater availability of vector breeding habitats and environmental conditions favorable to *Anopheles* mosquitoes [10,58,59]. However, these observations should be interpreted cautiously because the number of collected mosquitos was very low at certain sites, and CDC LT efficiency may vary according to ecological setting and mosquito species. Furthermore, mosquito collections at the urban La Voix du Peuple HA were limited, partly due to COVID-19-related movement restrictions preventing the completion of some collection rounds, potentially reducing the precision and representativeness of entomological estimates. Therefore, the findings from La Voix du Peuple may not necessarily indicate an absence of malaria transmission.

Insecticide resistance adds another layer of complexity to vector-control efforts in these study areas and across the DRC. Consistent with national trends, our findings reveal high resistance to pyrethroids and the near fixation of the L1014F *kdr* mutation in both Bu and Kimpoko HAs. This poses a significant threat to the effectiveness of pyrethroid-only LLINs, which remain the primary type of bed nets distributed in the study area [6,7,40,42,50]. These findings highlight the potential value of next-generation LLINs, including dual-active ingredient nets, in areas where pyrethroid resistance is widespread. Although large-scale IRS is not yet routinely implemented by the National Malaria Control Program in the DRC, the recent interest in developing national IRS guidelines highlights a growing focus on complementary vector-control approaches. In this context, the confirmed susceptibility of mosquito populations to bendiocarb and pirimiphos-methyl, insecticides commonly used in IRS programs, may provide valuable alternatives for future resistance-management and vector-control strategies.

Mutations in the voltage-gated sodium channel represent one of the principal mechanisms underlying resistance to pyrethroids and DDT in mosquito vectors [13,44,45]. While resistance-associated *kdr* mutations have been predominantly described in members of the *An. gambiae* complex, accumulating evidence indicates that similar target-site resistance mechanisms are also emerging in other mosquito species and genera, including *An. funestus*, *An. ziemanni*, *An. pharoensis*, and *Aedes aegypti* [27–29]. Notably, these mutations do not necessarily involve the same amino acid substitutions, reflecting the increasing heterogeneity and evolutionary diversification of insecticide resistance mechanisms in mosquito populations [29].

We observed an increase in mosquito mortality to DDT between 2019 and 2022. This unexpected trend is intriguing, especially considering that *Anopheles* populations have shown high resistance to DDT in most prior studies in the DRC [6,7,12,50]. A possible explanation is that DDT has not been in active use in routine vector control or agriculture for many years, thereby reducing the selective pressure that initially drove resistance. This trend may also reflect sampling variation, assay variability, or other unmeasured ecological and operational factors. Increases in mortality, although less pronounced, were also observed for other insecticides. Further longitudinal studies are needed to better understand the temporal dynamics and drivers of insecticide resistance in malaria vector populations in the DRC.

### Strengths and limitations

This is the first longitudinal entomological study conducted within the framework of a malaria cohort study in Kinshasa Province in the DRC. It provides longitudinal data on vector ecology, behavior, transmission intensity, and insecticide resistance across contrasting ecological settings, although several limitations should be acknowledged. First, funding constraints limited sampling to five households per study site per round, limiting our ability to detect household-level variations. In addition, the relatively small number of mosquitoes collected in some ecological settings (Pema and Voix du Peuple) may have reduced the precision of comparisons in mosquito abundance and estimated transmission intensity across sites and HAs. Specifically, the study design employed equal sampling across sites and only included one urban site, La Voix du Peuple HA, where mosquito populations may have been lower. Low sampling was further impacted by COVID-19-related movement restrictions preventing the completion of some collection rounds in this setting. Consequently, these disruptions further limited the precision and representativeness of entomological estimates, reducing the robustness of comparisons with peri-urban and rural settings.

Second, using CDC LTs as proxies for human biting rates may have introduced measurement biases, as their performance may vary according to mosquito species, density, ecological setting, and household characteristics. Additionally, LTs may attract or divert host-seeking mosquitoes differently in indoor and outdoor environments, potentially influencing observed densities and species composition across sites [31,33,35]. Consequently, these collections obtained using CDC LTs may not fully reflect true human biting behavior or direct human biting rates across settings. Furthermore, the sampling was grouped into two broad periods (before and after 10 PM) rather than conducted hourly, limiting fine-scale characterization of mosquito biting rhythms throughout the night.

Third, although collections occurred in both wet and dry seasons, calculating annual EIR by multiplying the daily values by 365 may overestimate transmission by not fully accounting for seasonal variation. Continuous year-round sampling would improve accuracy. In addition, sporozoite detection relied on CSP-ELISA, which is subject to false-positive and false-negative results. The former can occur because of cross-reacting heat-labile antigens, while the latter may arise from insufficient head-thorax material available for analysis [22–24]. Although standard laboratory procedures were followed, these methodological limitations should be considered when interpreting sporozoite rate estimates.

Fourth, at the time of testing, the correct dose of pirimiphos-methyl (0.25%) was used, however; the WHO updated the recommended dose in 2022 [60]. Future susceptibility testing should follow the revised protocol to ensure comparability and accuracy. Chlorfenapyr susceptibility was not evaluated, which represents an important gap given the increased deployment of chlorfenapyr-based LLINs in the country. In addition, *kdr* mutation testing was not performed after insecticide bioassays, preventing direct assessment of phenotypic-genotypic resistance linkage. Additionally, larval and household sampling were not always aligned.

## Conclusions

This entomological study highlights persistent malaria transmission and widespread pyrethroid resistance across diverse ecological settings in Kinshasa Province. The predominance of *An. gambiae* s.l., together with its largely indoor and late-night biting behavior, supports the continued relevance of LLIN-based interventions. However, the occurrence of early-evening and outdoor biting suggests potential gaps in protection that may contribute to residual malaria transmission. Additionally, the detection of high pyrethroid resistance, coupled with the near fixation of the L1014F *kdr* mutation, further emphasizes the need for resistance-informed vector control strategies and a move to dual action LLINs or additional vector control interventions. Nevertheless, preserved susceptibility to bendiocarb and pirimiphos-methyl provides promising opportunities for complementary interventions such as indoor residual spraying. The findings also suggest a possible localized contribution of *An. paludis* to malaria transmission, although additional studies are needed to better define its epidemiological importance. Overall, these findings underscore the importance of adaptive, locally informed malaria control strategies supported by sustained entomological surveillance to monitor vector behavior, transmission dynamics, and insecticide resistance across ecological settings in the DRC.

## Supporting information

S1 Table Mosquito collection schedule. (DOCX)

S2 Table Primers used in the study. (DOCX)

S3 Table *Anopheles* abundance across sites. (XLSX)

S1 Text DNA extraction protocol. (DOCX)

## References

[1] World Health Organization. World malaria report 2025. Geneva: WHO; 2025.

[2] Kang YO, Yabar H, Mizunoya T, Higano Y. Optimal landfill site selection using ArcGIS Multi-Criteria Decision-Making (MCDM) and Analytic Hierarchy Process (AHP) for Kinshasa City. Environmental Challenges. 2024;14:100826. doi: 10.1016/j.envc.2023.100826

[3] Karch PS, Asidi N, Manzambi ZM, Salaun JJ. La faune anophélienne à Kinshasa (Zaïre) et la transmission du paludisme humain. Bull Soc Pathol Exot. 1992; 85:123–9. 1446181

[4] Karch PS, Asidi N, Manzambi ZM, Salaun JJ. La faune culicidienne et sa nuisance à Kinshasa (Zaïre). Bull Soc Pathol Exot. 1993; 86:231–6. 8504267

[5] Coene J. Malaria in urban and rural Kinshasa: The entomological input. Med Vet Entomol. 1993;7(2):127–37. doi: 10.1111/j.1365-2915.1993.tb00665.x 8481529

[6] Riveron JM, Watsenga F, Irving H, Irish SR, Wondji CS. High plasmodium infection rate and reduced bed net efficacy in multiple insecticide-resistant malaria vectors in Kinshasa, Democratic Republic of Congo. J Infect Dis. 2018;217(2):320–8. doi: 10.1093/infdis/jix570 29087484 PMC5853898

[7] Nguiffo-Nguete D, Mugenzi LMJ, Manzambi EZ, Tchouakui M, Wondji M, Tekoh T, et al. Evidence of intensification of pyrethroid resistance in the major malaria vectors in Kinshasa, Democratic Republic of Congo. Sci Rep. 2023;13(1):14711. doi: 10.1038/s41598-023-41952-2 37679465 PMC10484898

[8] Wat’senga F, Manzambi EZ, Lunkula A, Mulumbu R, Mampangulu T, Lobo N, et al. Nationwide insecticide resistance status and biting behaviour of malaria vector species in the Democratic Republic of Congo. Malar J. 2018;17(1):129. doi: 10.1186/s12936-018-2285-6 29580247 PMC5870394

[9] Kashamuka MM, Banek K, White SJ, Atibu JL, Mvuama NM, Bala JAM, et al. Baseline characteristics for phase II of the Kinshasa Malaria Cohort Study: Cohort profile. BMJ Open. 2024;14(11):e085360. doi: 10.1136/bmjopen-2024-085360 39609012 PMC11603822

[10] Ferrari G, Ntuku HM, Schmidlin S, Diboulo E, Tshefu AK, Lengeler C. A malaria risk map of Kinshasa, Democratic Republic of Congo. Malar J. 2016;15:27. doi: 10.1186/s12936-015-1074-8 26762532 PMC4712518

[11] République Démocratique du Congo, Ministère de la Santé Publique, Programme National de Lutte contre le Paludisme. Plan stratégique national 2016–2020. Kinshasa; 2016.

[12] U.S. President’s Malaria Initiative. Democratic Republic of the Congo malaria operational plan FY 2023 [Internet]. 2023. https://www.pmi.gov

[13] Gatton ML, Chitnis N, Churcher T, Donnelly MJ, Ghani AC, Godfray HCJ, et al. The importance of mosquito behavioural adaptations to malaria control in Africa. Evolution. 2013;67(4):1218–30. doi: 10.1111/evo.12063 23550770 PMC3655544

[14] Bhatt S, Weiss DJ, Cameron E, Bisanzio D, Mappin B, Dalrymple U, et al. The effect of malaria control on Plasmodium falciparum in Africa between 2000 and 2015. Nature. 2015;526(7572):207–11. doi: 10.1038/nature15535 26375008 PMC4820050

[15] Frontline Health Project. The community health system in the DRC: An overview. Washington (DC): Population Council; 2020. https://knowledgecommons.popcouncil.org/departments_sbsr-rh/113/

[16] Mwandagalirwa MK, Levitz L, Thwai KL, Parr JB, Goel V, Janko M, et al. Individual and household characteristics of persons with Plasmodium falciparum malaria in sites with varying endemicities in Kinshasa Province, Democratic Republic of the Congo. Malar J. 2017;16(1):456. doi: 10.1186/s12936-018-2429-8 PMC5680818 29121931

[17] Gillies MT, De Meillon B. The Anophelinae of Africa south of the Sahara (Ethiopian Zoogeographical Region). 2nd ed. Johannesburg: South African Institute for Medical Research; 1968. 343.

[18] Coetzee M. Key to the females of Afrotropical Anopheles mosquitoes (Diptera: Culicidae). Malar J. 2020;19(1):70. doi: 10.1186/s12936-020-3144-9 32054502 PMC7020601

[19] World Health Organization. Test procedures for insecticide resistance monitoring in malaria vector mosquitoes. Geneva: WHO; 2016.

[20] Salako AS, Ossè R, Padonou GG, Dagnon F, Aïkpon R, Kpanou C, et al. Population dynamics of *Anopheles gambiae* s.l. and Culex quinquefasciatus in rural and urban settings before an indoor residual spraying campaign in northern Benin. Vector Borne Zoonotic Dis. 2019;19(9):674–84. doi: 10.1089/vbz.2018.2409 30964413 PMC6716193

[21] Santolamazza F, Mancini E, Simard F, Qi Y, Tu Z, della Torre A. Insertion polymorphisms of SINE200 retrotransposons within speciation islands of Anopheles gambiae molecular forms. Malar J. 2008;7:163. doi: 10.1186/1475-2875-7-163 18724871 PMC2546427

[22] Wirtz RA, Burkot TR, Andre RG, Rosenberg R, Collins WE, Roberts DR. Identification of Plasmodium vivax sporozoites in mosquitoes using an enzyme-linked immunosorbent assay. Am J Trop Med Hyg. 1985;34(6):1048–54. doi: 10.4269/ajtmh.1985.34.1048 2422965

[23] De Arruda ME, Collins KM, Hochberg LP, Ryan PR, Wirtz RA, Ryan JR. Quantitative determination of sporozoites and circumsporozoite antigen in mosquitoes infected with Plasmodium falciparum or P. vivax. Ann Trop Med Parasitol. 2004;98(2):121–7. doi: 10.1179/000349804225003181 15035722

[24] Beier JC, Asiago CM, Onyango FK, Koros JK. ELISA absorbance cut-off method affects malaria sporozoite rate determination in wild Afrotropical Anopheles. Med Vet Entomol. 1988;2(3):259–64. doi: 10.1111/j.1365-2915.1988.tb00193.x 2980182

[25] Huynh LY, Sandve SR, Hannan LM, Van Ert M, Gimnig JE. Fitness costs of pyrethroid insecticide resistance in *Anopheles gambiae*. Christchurch, New Zealand; 2007.

[26] Acford-Palmer H, Campos M, Bandibabone J, N’Do S, Bantuzeko C, Zawadi B, et al. Detection of insecticide resistance markers in Anopheles funestus from the Democratic Republic of the Congo using a targeted amplicon sequencing panel. Sci Rep. 2023;13(1):17363. doi: 10.1038/s41598-023-44457-0 37833354 PMC10575962

[27] Odero JO, Dennis TPW, Polo B, Nwezeobi J, Boddé M, Nagi SC, et al. Discovery of knock-down resistance in the major african malaria vector *Anopheles funestus*. Mol Ecol. 2024;33(22):e17542. doi: 10.1111/mec.17542 39374937 PMC11537839

[28] Mayi MPA, Antonio-Nkondjio C, Bamou R, Damiani C, Cappelli A, Djiappi-Tchamen B, et al. First detection of kdr L1014F allele in *Anopheles ziemanni* and *Anopheles pharoensis* in Cameroon and distribution of the allele in members of the *Anopheles gambiae* complex. Parasit Vectors. 2024;17(1):363. doi: 10.1186/s13071-024-06420-4 39192348 PMC11348551

[29] Du Y, Nomura Y, Zhorov BS, Dong K. Sodium channel mutations and pyrethroid resistance in *Aedes aegypti*. Insects. 2016;7(4):60. doi: 10.3390/insects7040060 27809228 PMC5198208

[30] Pan W. Akaike’s information criterion in generalized estimating equations. Biometrics. 2001;57(1):120–5. doi: 10.1111/j.0006-341x.2001.00120.x 11252586

[31] Lines JD, Curtis CF, Wilkes TJ, Njunwa KJ. Monitoring human-biting mosquitoes (Diptera: Culicidae) in Tanzania with light traps hung beside mosquito nets. Bull Entomol Res. 1991;81(1):77–84. doi: 10.1017/S0007485300053268

[32] Namango IH, Marshall C, Saddler A, Ross A, Kaftan D, Tenywa F, et al. The CDC light trap and the human decoy trap compared to the human landing catch for measuring *Anopheles* biting in rural Tanzania. Malar J. 2022;21(1):181. doi: 10.1186/s12936-022-04192-9 PMC9188237 35690745

[33] Overgaard HJ, Saebø S, Reddy MR, Reddy VP, Abaga S, Matias A, et al. Light traps fail to estimate reliable malaria mosquito biting rates on Bioko Island, Equatorial Guinea. Malar J. 2012;11:56. doi: 10.1186/1475-2875-11-56 22364588 PMC3384454

[34] Magbity EB, Magbity EB, Lines JD, Marbiah MT, David K, Peterson E. How reliable are light traps in estimating biting rates of adult Anopheles gambiae s.l. (Diptera: Culicidae) in the presence of treated bed nets?. Bull Entomol Res. 2002;92(1):71–6. doi: 10.1079/BER2001131 12020364

[35] Briët OJT, Huho BJ, Gimnig JE, Bayoh N, Seyoum A, Sikaala CH, et al. Applications and limitations of Centers for Disease Control and Prevention miniature light traps for measuring biting densities of African malaria vector populations: A pooled-analysis of 13 comparisons with human landing catches. Malar J. 2015;14:247. doi: 10.1186/s12936-015-0761-9 26082036 PMC4470360

[36] Dida GO, Anyona DN, Abuom PO, Akoko D, Adoka SO, Matano A-S, et al. Spatial distribution and habitat characterization of mosquito species during the dry season along the Mara River and its tributaries, in Kenya and Tanzania. Infect Dis Poverty. 2018;7(1):2. doi: 10.1186/s40249-017-0385-0 29343279 PMC5772712

[37] Metelo E, Bukaka E, Luemba TB, Situakibanza H, Sangaré I, Mesia G, et al. Détermination des paramètres bioécologiques et entomologiques d’Anopheles gambiae sl dans la transmission du paludisme à Bandundu-ville, République Démocratique de Congo. Pan Afr Med J. 2015;22. doi: 10.11604/pamj.2015.22.108.6774 PMC4732634 26848355

[38] Kahamba NF, Tarimo FS, Kifungo K, Mponzi W, Kinunda SA, Simfukwe A, et al. Societal uses of the main water bodies inhabited by malaria vectors and implications for larval source management. Malar J. 2024;23(1):336. doi: 10.1186/s12936-024-05154-z 39521968 PMC11550540

[39] Akuoko OK, Dhikrullahi SB, Hinne IA, Mohammed AR, Owusu-Asenso CM, Coleman S, et al. Biting behaviour, spatio-temporal dynamics, and the insecticide resistance status of malaria vectors in different ecological zones in Ghana. Parasit Vectors. 2024;17(1):16. doi: 10.1186/s13071-023-06065-9 38195546 PMC10775458

[40] Zanga J, Metelo E, Mvuama N, Nsabatien V, Mvudi V, Banzulu D, et al. Species composition and distribution of the *Anopheles gambiae* complex circulating in Kinshasa. GigaByte. 2024; 2023:1–12. doi: 10.46471/gigabyte104 PMC10777374 38213983

[41] Sinka ME, Bangs MJ, Manguin S, Coetzee M, Mbogo CM, Hemingway J, et al. The dominant Anopheles vectors of human malaria in Africa, Europe and the Middle East: occurrence data, distribution maps and bionomic précis. Parasit Vectors. 2010;3:117. doi: 10.1186/1756-3305-3-117 21129198 PMC3016360

[42] Zanga J, Metelo E, Mbanzulu K, Irish S, Mulenda B, Di-Mosi W, et al. Susceptibility status of *Anopheles gambiae* s.l. to insecticides used for malaria control in Kinshasa, Democratic Republic of the Congo. Ann Afr Med. 2022;15(2):4533. Available from: 10.4314/aam.v15i2.2

[43] Metelo E, Zanga J, Batumbo D, Mandja BA, Lukoki H, Bokulu A, et al. Complexity of vector control and entomological surveillance in endemic sentinel sites of the National Malaria Control Program (NMCP) in the Democratic Republic of the Congo (DRC). 2024. https://www.intechopen.com

[44] Sherrard-Smith E, Skarp JE, Beale AD, Fornadel C, Norris LC, Moore SJ, et al. Mosquito feeding behavior and how it influences residual malaria transmission across Africa. Proc Natl Acad Sci U S A. 2019;116(30):15086–95. doi: 10.1073/pnas.1820646116 31285346 PMC6660788

[45] Sougoufara S, Ottih EC, Tripet F. The need for new vector control approaches targeting outdoor biting Anopheline malaria vector communities. Parasit Vectors. 2020;13(1):295. doi: 10.1186/s13071-020-04170-7 32522290 PMC7285743

[46] Githeko AK, Adungo NI, Karanja DM, Hawley WA, Vulule JM, Seroney IK, et al. Some observations on the biting behavior of Anopheles gambiae s.s., Anopheles arabiensis, and Anopheles funestus and their implications for malaria control. Exp Parasitol. 1996;82(3):306–15. doi: 10.1006/expr.1996.0038 8631382

[47] Bobanga T, Umesumbu SE, Mandoko AS, Nsibu CN, Dotson EB, Beach RF, et al. Presence of species within the Anopheles gambiae complex in the Democratic Republic of Congo. Trans R Soc Trop Med Hyg. 2016;110(6):373–5. doi: 10.1093/trstmh/trw035 27317755

[48] Sharma A, Kinney NA, Timoshevskiy VA, Sharakhova MV, Sharakhov IV. Structural Variation of the X Chromosome Heterochromatin in the Anopheles gambiae Complex. Genes (Basel). 2020;11(3):327. doi: 10.3390/genes11030327 32204543 PMC7140835

[49] Bobanga T, Ayieko W, Zanga M, Umesumbu S, Landela A, Fataki O, et al. Field efficacy and acceptability of PermaNet® 3.0 and OlysetNet® in Kinshasa, Democratic Republic of the Congo. J Vector Borne Dis. 2013;50(3):206–14. doi: 10.4103/0972-9062.120929 24220080

[50] Wat’senga F, Agossa F, Manzambi EZ, Illombe G, Mapangulu T, Muyembe T, et al. Intensity of pyrethroid resistance in Anopheles gambiae before and after a mass distribution of insecticide-treated nets in Kinshasa and in 11 provinces of the Democratic Republic of Congo. Malar J. 2020;19(1):169. doi: 10.1186/s12936-020-03240-6 32354333 PMC7193383

[51] Metelo-Matubi E, Zanga J, Binene G, Mvuama N, Ngamukie S, Nkey J, et al. The effect of a mass distribution of insecticide-treated nets on insecticide resistance and entomological inoculation rates of *Anopheles gambiae* s.l. in Bandundu City, Democratic Republic of the Congo. Pan Afr Med J. 2021;40:1–10. doi: 10.11604/pamj.2021.40.118.27365 PMC8627145 34887992

[52] De Marco CM, Virgillito C, Frosi L, Santarelli G, Filipponi F, Manica M, et al. Habitat drivers and predicted distribution shifts of *Anopheles coluzzii* under climate change: Results from the systematic review. Sci Total Environ. 2025;992:179939. doi: 10.1016/j.scitotenv.2025.179939 40582050

[53] Longo-Pendy NM, Tene-Fossog B, Tawedi RE, Akone-Ella O, Toty C, Rahola N, et al. Ecological plasticity to ions concentration determines genetic response and dominance of *Anopheles coluzzii* larvae in urban coastal habitats of Central Africa. Sci Rep. 2021;11(1):15781. doi: 10.1038/s41598-021-94258-6 34349141 PMC8338965

[54] Costantini C, Ayala D, Guelbeogo WM, Pombi M, Some CY, Bassole IH, et al. Living at the edge: Biogeographic patterns of habitat segregation conform to speciation by niche expansion in Anopheles gambiae. BMC Ecol. 2009;9:16. doi: 10.1186/1472-6785-9-16 19460144 PMC2702294

[55] Diabaté A, Dabiré RK, Heidenberger K, Crawford J, Lamp WO, Culler LE, et al. Evidence for divergent selection between the molecular forms of Anopheles gambiae: Role of predation. BMC Evol Biol. 2008;8:5. doi: 10.1186/1471-2148-8-5 18190719 PMC2217532

[56] Diabaté A, Dabire RK, Kim EH, Dalton R, Millogo N, Baldet T, et al. Larval development of the molecular forms of Anopheles gambiae (Diptera: Culicidae) in different habitats: A transplantation experiment. J Med Entomol. 2005;42(4):548–53. doi: 10.1093/jmedent/42.4.548 16119542

[57] Carrel M, Kim S, Mwandagalirwa MK, Mvuama N, Bala JA, Nkalani M, et al. Individual, household and neighborhood risk factors for malaria in the Democratic Republic of the Congo support new approaches to programmatic intervention. Health Place. 2021;70:102581. doi: 10.1016/j.healthplace.2021.102581 34020231 PMC8328915

[58] Sendor R, Banek K, Kashamuka MM, Mvuama N, Bala JA, Nkalani M, et al. Epidemiology of Plasmodium malariae and Plasmodium ovale spp. in a highly malaria-endemic country: A longitudinal cohort study in Kinshasa Province, Democratic Republic of the Congo. Malar J. 2023;22(1):97. doi: 10.1101/2023.04.20.2328882636932389

[59] Diallo AO, Banek K, Kashamuka MM, Bala JAM, Nkalani M, Kihuma G, et al. Impact of malaria diagnostic choice on monitoring of Plasmodium falciparum prevalence estimates in the Democratic Republic of the Congo and relevance to control programs in high-burden countries. PLOS Glob Public Health. 2023;3(7):e0001375. doi: 10.1371/journal.pgph.0001375 37494361 PMC10370698

[60] World Health Organization (WHO). WHO guidelines for malaria. Geneva: World Health Organization; 2022. https://www.who.int/publications/i/item/guidelines-for-malaria

