# Supplementary material for: Malaria entomological surveillance in Kinshasa Province: A longitudinal study
Source: PLOS Glob Public Health. 2026 Aug 5;6(8):e0006984. doi: 10.1371/journal.pgph.0006984 (PMC13440810; doi:10.1371/journal.pgph.0006984)
Supplement: Supplementary file 1 [file pgph.0006984.s001.docx]

**Mosquito collection schedule**

| **Year** | **Collection round** | **Period** | **Season** |
| --- | --- | --- | --- |
| 2018 | Round 1 | March 2018 | Rainy |
|  | Round 2 | July – August 2018 | Dry |
|  | Round 3 | December 2018 | Rainy |
| 2019 | Round 4 | April – May 2019 | Rainy |
|  | Round 5 | August 2019 | Dry |
|  | Round 6 | December 2019 | Rainy |
| 2020 | Round 7 | March 2020 | Rainy |
|  | Round 8 | August 2020 | Dry |
| 2021 | Round 9 | March 2021 | Rainy |
