# Supplementary material for: Malaria entomological surveillance in Kinshasa Province: A longitudinal study
Source: PLOS Glob Public Health. 2026 Aug 5;6(8):e0006984. doi: 10.1371/journal.pgph.0006984 (PMC13440810; doi:10.1371/journal.pgph.0006984)
Supplement: Supplementary file 2 [file pgph.0006984.s002.docx]

1. **Primers used in PCR analysis**

| **Analysis** | **Primers** | **Sequences (5’ → 3’)** |
| --- | --- | --- |
| *Anopheles gambiae* sibling species | F6 | TCGCCTTAGACCTTGCGTTA |
|  | R5 | CGCTTCAAGAATTCGAGATAC |
| L1014F *kdr* mutation | IPCF | GATAATGTGGATAGATTCCCCGACCATG |
|  | AltRev | TGCCGTTGGTGCAGACAAGGATG |
|  | West WT | GGTCCATGTTAATTTGCATTACTTACGAATA |
|  | West F | CTTGGCCACTGTAGTGATAGGAAAATC |

2. **PCR mixture and conditions for molecular identification of sibling species of the *Anopheles gambiae* complex**

The 25 µl reaction mixture consisted of 13.25 µl of PCR-grade water, 5 µl of 5x buffer, 1 µl of 5 mM dNTPs, 1.5 µl of MgCl2 25mM, 1 µl of the F6 primer, 1 µl of the R5 primer, 0.25 µl of Taq polymerase, and 2 µl of DNA template. The PCR conditions were as follows: initial denaturation at 94 °C for 3 minutes, followed by 35 cycles of denaturation at 94 °C for 30 seconds, annealing at 56 °C for 30 seconds, elongation at 72 °C for 30 seconds, and a final elongation cycle at 72°C for 5 minutes. The amplified products, corresponding to different sibling species, were separated by 2% agarose gel electrophoresis.

The 25 µl reaction mixture consisted of 11.3 µl PCR-grade water, 5 µl of 5x buffer, 1 µl of 5 mM dNTPs, 1.5 µl of 25 mM MgCl2, 1 µl each of IPCF, AltRev, West WT, and West F primers, 0.2 µl of Taq polymerase, and 2 µl of DNA template (Supporting information 2). The PCR conditions were as follows: an initial denaturation at 95 °C for 5 minutes, followed by 35 cycles of denaturation at 95 °C for 30 seconds, annealing at 59 °C for 30 seconds, elongation at 72 °C for 30 seconds, and a final elongation at 72°C for 30 seconds. The amplified products, representing different genotypes, were separated by 2% agarose gel electrophoresis.
