# Supplementary material for: Malaria entomological surveillance in Kinshasa Province: A longitudinal study
Source: PLOS Glob Public Health. 2026 Aug 5;6(8):e0006984. doi: 10.1371/journal.pgph.0006984 (PMC13440810; doi:10.1371/journal.pgph.0006984)
Supplement: Supplementary file 3 [file pgph.0006984.s004.docx]

**Supporting information 1: DNA Extraction Protocol**

1) Grind each organ in 200 µl of 2% CTAB;

2) Place in a heating block at 65°C for 5 minutes;

3) Add 200 µl of chloroform (use the same cone as long as it hasn't touched the tubes) and mix by inversion at least 10 times;

4) Centrifuge for 5 minutes at 12,000 rpm at room temperature;

5) Prepare corresponding tubes, well-labeled, to collect the supernatant;

6) Retrieve the upper phase (supernatant) and place it in the prepared tubes;

7) Add 200 µl of isopropanol to the supernatant and mix by inversion;

8) Centrifuge for 10 minutes at 12,000 rpm at room temperature;

9) Discard the isopropanol, drain well, and add 200 µl of 70% ethanol;

10) Centrifuge for 5 minutes at 12,000 rpm at room temperature;

11) Discard the ethanol and dry the DNA pellet for 5 minutes under speed-vac;

12) Resuspend in 20 µl of sterile H2O (use a new cone for each tube) - Leave to suspend overnight on the bench or for half a day;

13) Make the appropriate dilutions for PCRs.
